# Driving under the influence of cannabis: perceptions from Canadian youth

**DOI:** 10.1186/s12889-022-14658-9

**Published:** 2022-12-19

**Authors:** Jennifer R. Donnan, Dalainey H. Drakes, Emily C. Rowe, Maisam Najafizada, Lisa D. Bishop

**Affiliations:** 1 School of Pharmacy, Memorial University of Newfoundland, 300 Prince Philip Drive, St. John’s, NL A1B 3V6 Canada; 2 Department of Psychology, Memorial University of Newfoundland, St. John’s, NL Canada; 3 Faculty of Medicine, Memorial University of Newfoundland, St. John’s, NL Canada

**Keywords:** Cannabis, Canada, Driving, Substance use, Youth, Young adults

## Abstract

**Background:**

Cannabis legalization is intended to protect the public from potential harm by restricting access and promoting greater awareness of cannabis-related risks. Youth are at a greater risk for experiencing road-related harms due to their own or others’ use of cannabis. This qualitative research explored youths’ perceptions about cannabis and road safety.

**Methods:**

A qualitative study using focus groups (FG) was conducted with youth (age 13-18) and young adults (age 19-25) who resided in Newfoundland and Labrador. Using semi-structured interview questions, the facilitator asked participants to share their opinions about cannabis and road safety. All sessions were hosted virtually using *Zoom* with recruitment until saturation was met. All sessions were audio recorded, de-identified, and transcribed. Analysis utilized an inductive thematic approach informed by Braun and Clarke’s (2006) method and inductive coding was facilitated using *NVivo*.

**Results:**

Six youth (*n =* 38) and five young adult (*n =* 53) FG were conducted. Five prominent themes emerged throughout discussions across both age groups including: a) normalization of driving under the influence of cannabis, b) knowledge and awareness, c) perceptions of risk, d) modes of transportation, and e) detection. Variation in perceptions appeared to be influenced by lack of awareness of the impact of cannabis on driving ability, residence in urban versus rural locations, type of vehicle driven (e.g., car vs. off-road vehicles), and gender.

**Conclusion:**

The themes uncovered from this research will help inform future enhancement of cannabis policy to ensure the safety of all citizens. These findings will also support the inclusion of youth-focused education that will equip youth with informed decision-making strategies regarding road safety. Furthermore, these findings can be utilized to inform the refinement of cannabis driving policies to ensure the safety of all citizens on or off the road.

**Supplementary Information:**

The online version contains supplementary material available at 10.1186/s12889-022-14658-9.

## Background

The legalization of cannabis in Canada was intended to protect youth from potential harm; however, youth have been identified as the population at highest risk for experiencing threats to their health or safety due to their own or others’ use of cannabis while on the road [1, 2]. Cannabis use has been associated with changes in one’s driving ability and linked to specific vehicle operation behaviours such as reduced driving speed, slower reaction time, increased headway distance, higher frequency of lane changes, and overall diminished visual function [3–5]. Driving under the influence of cannabis (DUIC) has been identified as the most common potentially harmful cannabis consumption-related behaviour [3, 6]. Cannabis is one of the most common substances involved in car crashes and accounts for 20% of fatal DUI crashes [7, 8]. More specifically, recent national data supports that the highest prevalence of risk for engaging DUIC-related behaviours continues to be observed among Canadians aged 15 to 24 years of age [3, 9, 10]. Alarmingly high prevalence rates of Canadian youth cannabis consumers have been previously reported with upwards of 64% of males and 33% of females reporting having previously DUIC [11]. Youth DUIC pose additional road safety risks due to the combination of effects from intoxication and developmental factors. Youth are more likely to get distracted and be involved in distraction-related accidents [12, 13], this may be partially due to their inexperience in dividing their attention to the various aspects of driving [14]. The public safety concerns become even more pressing, with 80% of male and 75% female young cannabis consumers having been a passenger in a vehicle of a cannabis-impaired driver [11]. The prevalence of young driver engagement in DUIC or passengers impacted by DUIC is particularly concerning and youth non-consumers have a stake in the commonality of DUIC as passengers seeking transportation are at risk for DUI-related harms. Youth between 16 and 19 years of age are already at three times greater risk of involvement in fatal car accidents than adults 20 years and older [15]. Youth have consistently been found to engage in more reckless or risky driving behaviours. When coupled with substance use, the chance of harm to oneself, passengers, and others near the roadside is exacerbated [11, 16].

Cannabis-impairment tends to be strongest for non-regular consumers; however, frequent consumers are not immune to deficits exerted on one’s executive control over their cognitive and psychomotor responses [4, 5, 17]. Impairment can lead to deficits in cognitive abilities, including but not limited to attentional capacity, concentration, and decision making [5]. Consequently, individuals will have difficulty in emotional processing and motor response to stimuli on the road resulting in slow reactions imperative for operating motorized vehicles [18, 19].

Previous research has found that Canadian youth who chronically consume cannabis tend to be more willing to engage in risky behaviours than occasional users [20] and are more likely to be under the influence of multiple substances than only cannabis [21]. Youth who experienced early initiation before the age of 16 and who consume cannabis regularly have exhibited the worse driving ability and heightened impulsivity on the road [21]. As a result, it is essential to consider individual factors to reduce the prevalence of DUIC among young drivers, such as the age of initiation, sex, frequency of cannabis consumption, and personality factors.

Recent structural equation modelling of the DUIC belief system supported the notion that participation in DUIC is predicated on an interaction between an individual’s willingness and intention [22, 23]. Moreover, one’s DUIC-related beliefs are further promoted by the normative beliefs commonly upheld within a society [22, 23], acceptance by family, and approval by other consumers [24]. The act of DUIC has increasingly become a more socially acceptable behaviour due to its convenience [25] and belief that the relative risk of DUIC is lower than other substances [24] or dependent on cannabis use factors (i.e., tolerance, product choice, mode of consumption [26]). Perception of less harm has been associated with consumers more than non-consumers [27] and especially among Canadian youth [28] or adults [29].

This qualitative research explored youths’ perceptions of the impact of cannabis on road safety in Newfoundland and Labrador (NL). At the time this data was collected roadside drug screening devices that test for cannabis had just been obtained by police services in NL, however they were not in widespread use [30]. Testing at this time was limited to Standardized Field Sobriety Test or identification by a drug recognition expert. The purpose of the present study was to gather insights from youth residing in urban and rural communities on their perceptions of the impact of cannabis on road safety, based on their firsthand knowledge and experience. The primary objectives were to:

1. Gain a better understanding of how youth perceived driving under the influence of cannabis.
2. Explore factors that influenced impaired driving behaviour.
3. Gather insight on knowledge gaps about cannabis education and driving.

## Method

### Recruitment of participants

The recruitment of youth and young adults occurred between May and June 2021. Youth were invited to participate in a focus group (FG) if they: 1) were from the age of 13 to 18; 2) were a resident of NL; and 3) consented to participate (both youth and a parent or caregiver). Young adults were included if they: 1) were from the age of 19 to 25; 2) were a resident of NL; and 3) provided informed consent. Personal experience with cannabis was not a requirement to participate as non-consumers may have experiences involving DUIC or have directly witnessed behaviours (i.e., being a passenger of an impaired driver or having been a witness to peer consumption prior to operation of a vehicle). Targeted recruitment efforts were made across NL with consideration given to geography, sex/gender, and prior cannabis experience as DUI can be impactful for consumers as well as non-consumers. Calls for participants were facilitated through a variety of modalities to share information letters and consent materials, including a) correspondence with local youth programs, agencies, and the Government of NL; b) sharing recruitment advertisements via social media; c) features in newsletters across post-secondary institutions in NL; and d) snowball sampling.

### Data collection

**Fig. 1:**
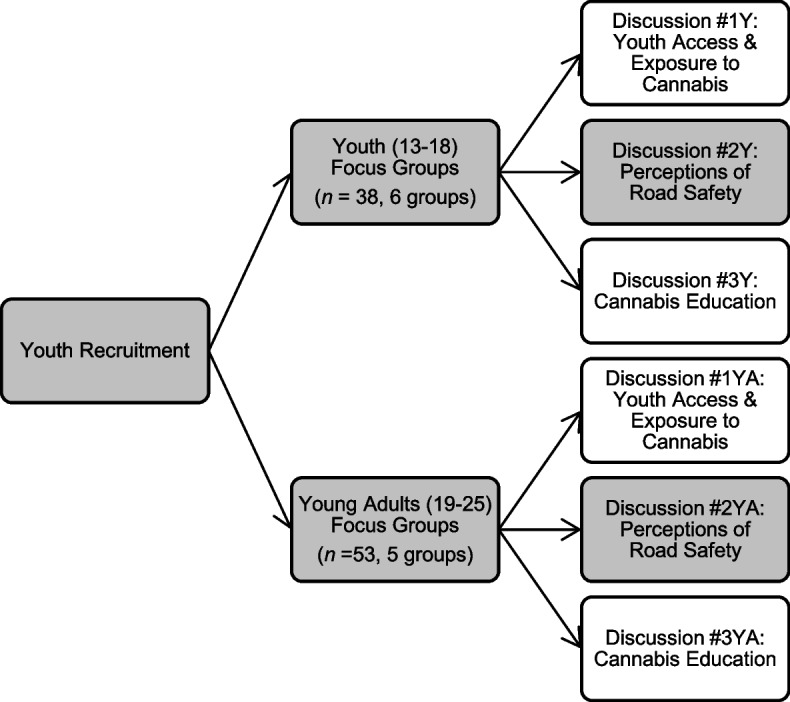
Focus group facilitation flowchart. Note. Y = youth, YA = young adult. Identified themes in the present qualitative study are from a larger series of discussions with youth, therefore, the shaded cells identify which focus groups and discussion topics are explored herein

Demographic information including age, gender, size of geographic location (e.g., urban, large rural, small rural), highest level of education, past receipt of cannabis education, and experience with cannabis consumption was collected. Focus groups were designed to be 2 h long and included three distinct discussion topics: access to cannabis, driving and cannabis, and cannabis education. Only data pertaining to the cannabis and driving discussion are reported in this manuscript (Fig. 1).

FGs were run exclusively with youth or young adults with no overlap across age groups and attendance ranged from 3 to 14 participants per session and were further divided into breakout rooms ranging in size from 3 to 7 participants. The discussion, led by D.H.D. and E.C.R., followed a semi-structured guide (see online supplement 1). Questions were typed into the chat and responses were read aloud for those who preferred to participate in the chat forum instead of speaking aloud. All sessions were audio-recorded, transcribed, and de-identified. Data collection continued until saturation was met and no new emerging themes were uncovered. All participants received a $50 gift card.

### Researcher Positionality

The research team on the present study was a gender-balanced, culturally diverse group inclusive of healthcare researchers in the disciplines of pharmacy, psychology, and medicine. The interview teams included a woman and man interviewer who were healthcare research trainees. All researchers and trainees were cautious of their healthcare related training and professional backgrounds; therefore, every effort was made to ensure this did not impact our data collection and qualitative analyses. The research team actively strived to maintain a neutral perspective with discussions guided by participant responses as opposed to any healthcare related objectives. All researchers and trainees recognized that healthcare professionals can be perceived as an individual of higher power than the general public, therefore, discussion with participants was solely facilitated by the trainees to mitigate any potential power imbalance.

### Thematic analysis

De-identified transcripts were coded using an inductive thematic analysis approach following the approach outlined by Braun and Clarke (2006) [31], wherein D.H.D. and E.C.R. independently analyzed two transcripts for consistency and reliability of inter-rater coding of the transcripts. *NVivo* software was utilized for the coding, storage, and organization of data. The coders developed a codebook with ongoing updates on emerging themes until no new ones were identified, and the other team members provided input into the identification of the overarching themes. D.H.D. and E.C.R coded the transcripts using an inductive line by line approach to coding. An inductive approach was completed as there is little knowledge presently known about the impact of cannabis legalization on youth and young adults’ perceptions of road safety in NL. Constant comparison was used to examine relationships between and across thematic codes or categories between coders to maintain consistency while discussing disagreements until consensus was reached. When reporting results below, quotes have been edited for clarity. To provide context to the participant demographic, quotes are identified by the gender and age group (youth or young adult) using the terms girl, woman, boy, man, transgender or non-binary as well as rural or urban dwelling status, cannabis consumption experience, and a unique study identification number (e.g., *Urban Girl Non-Consumer 25)*.

### Mapping of themes

Following completion of the inductive thematic analysis of transcripts, identified themes and sub-themes were further categorized into antecedents, choice points, and consequences associated with DUIC as informed by the multi-level and systems model of driving behaviour [32]. This conceptual framework explains how individual and contextual factors interact with behaviour-related decision making and future consequences of an individual’s actions. The model includes variables beyond the individual including sociocultural context, policies and regulations, transportation infrastructure, community education interventions, individual characteristics, road and weather conditions, and features of vehicles affecting the ultimate outcome of DUIC. The commonly reported perceptions speaking to antecedents, choice points, and consequences were then coded to identify potentially common factors related to DUIC.

## Results

### Study sample

This study recruited 91 participants between the age of 13 to 25 into six youth (*n* = 38) and five young adult (*n* = 53) FG (Table 1). The mean age of the youth sample was 15.4 (*SD* = 8.1) and the mean age of the young adult sample was 21.8 *(SD* = 1.9) with the majority of participants having self-identified as girls or women (70.3%). Representation of participants was diverse with respect to geography and prior experience with cannabis. An additional eight youth and ten young adults failed to attend their scheduled FG. All youth and young adult FG were on average 1.2 hours (*SD* = 0.3) in length ranging from 0.9 to 1.8 hours as guided by the conversation.

**Table 1:** Summary of youth and young adult sample characteristics

Demographic Variable	Youth *n*(%)	Young Adults *n*(%)	Total *n*(%)
Number of Focus Groups	6	5	11
Number of Participants	38	53	91
Education			
Grade 7-9	17 (44.7)	…	17 (18.7)
Grade 10-12	14 (36.8)	3 (5.7)	17 (18.7)
Some Post-Secondary	6 (15.8)	26 (49.1)	32 (35.2)
College Diploma	1 (2.6)	7 (13.2)	8 (8.8)
Bachelor’s Degree	…	14 (26.4)	14 (15.4)
Graduate Degree	…	3 (5.7)	3 (3.2)
Geographic Location			
Small Rural (less than 5000)	11 (28.9)	6 (11.3)	17 (18.7)
Large Rural (5000 – 30,000)	5 (13.2)	15 (28.3)	20 (22.0)
Urban (30,000 plus)	22 (57.9)	32 (60.4)	54 (59.3)
Age			
13-15	20 (52.6)	…	20 (22.0)
16-18	18 (47.4)	…	18 (19.8)
19-21	…	29 (54.7)	29 (31.9)
22-25	…	24 (45.3)	24 (26.3)
Gender			
Boy or Man	10 (26.3)	15 (28.3)	25 (27.5)
Girl or Woman	27 (71.1)	37 (69.8)	64 (70.3)
Transgender	1 (2.6)	…	1 (1.1)
Non-Binary	…	1 (1.9)	1 (1.1)
Previous Cannabis Education			
Yes	21 (55.3)	35 (66.1)	56 (61.5)
No	11 (28.9)	14 (26.4)	25 (27.5)
Unsure	6 (15.8)	4 (7.5)	10 (11.0)
Cannabis Consumption			
Regular Consumption^a^	7 (18.4)	20 (37.7)	27 (29.7)
Consumption in Past 3 Months	3 (7.9)	8 (15.1)	11 (12.1)
Consumption in Past Year	…	10 (18.9)	10 (11.0)
Never Consumed	27 (71.1)	13 (24.5)	40 (43.9)
Prefer Not to Say	1 (2.6)	2 (3.8)	3 (3.3)

^a^ at least once per month

### Thematic analysis

**Fig. 2:**
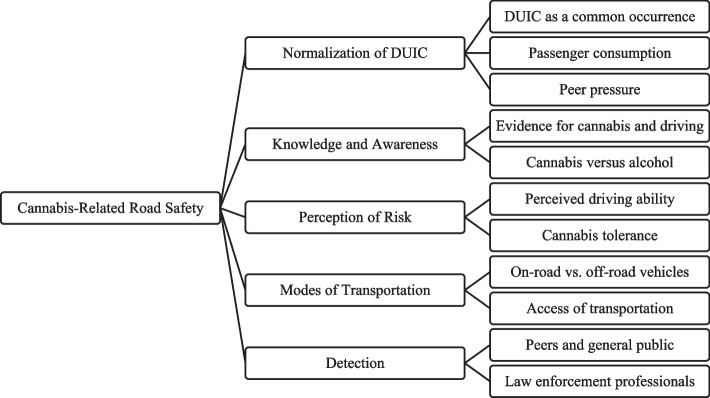
Emerging road safety themes and sub-themes

The findings from thematic analysis of discussions pertaining to road safety are described herein. Five main themes across age groups emerged including: 1) normalization of DUIC; 2) knowledge and awareness; 3) perceptions of risk; 4) modes of transportation and 5) detection (see Fig. 2).

#### Theme 1: normalization of DUIC

Many participants commented that DUIC had become a normalized part of society. This was in relation to: i) DUIC as a common occurrence, ii) passenger consumption, and iii) peer pressure.

i). DUIC as a common occurrence.

Youth and young adults mentioned that they commonly witness people driving after consuming cannabis, especially since cannabis consumption has become legal. As noted by one youth,

*People aren’t hiding the fact that they’re using marijuana as much where it’s legal. And you’ve definitely seen a lot more people out on the roads … you smell it a lot more, and it’s not just in the shady parking lot or somewhere like a backroad on the way to the cabin. (Urban Girl Non-Consumer 60).*

Another youth mentioned that because smoking cannabis and driving is so common, DUIC has become increasingly perceived as the norm:

*if you drive drunk, you’re an ass, like no one will disagree with that. But if you smoke, if you smoke and drive like it’s so normalized, everyone does it. People you like do it. Your friends are doing it. (Urban Girl Non-Consumer 25).*

The frequency of cannabis-impaired driving was also believed to be the more common among young boysand men than girls or women. Personality differences were attributed as the main contributors associated with boy/men engagement in more reckless behaviour, waiting a shorter interval after consumption, and the tendency to be more ill-prepared than girls/women who more cautiously plan in advance.

*I find girls are more prepared when they’re about to smoke, like they have a ride home and they know where they’re going to do it and they know who they’re doing it with and they know where they got it from. Boys usually take their car and they go drive... Then, they smoke and drive somewhere else, pick up their friends, they smoke again and then go home. So I think that they don’t really think about, like the dangers of it. (Urban Girl Non-Consumer 25).*

ii). Passenger consumption

It was discussed during some FG sessions that “*Sometimes it’s not the person who’s driving, it’s just the other people that they have in their car.” (Urban Girl Consumer 99).*

It was also noted that driving was not always just a means of transportation, but that driving in-and-of-itself was a common activity, and smoking cannabis while driving has become a part of that social activity. Some participants discussed how passengers readily consumed cannabis in the vehicles, on their own, or with the driver.

*if there’s nothing to be at, people just kind of drive around and then it’s like maybe if you’re with someone and they’re smoking and then it’s kind of, oh, I’ll just have one puff or just a few and then it kind of spirals that way. (Rural Woman Consumer 66).*

Some suggested that passenger consumption can be dependent on the rules of the driver, as one person stated *“I’ve never smoked weed in a car or had someone else smoke and I think for me personally, I wouldn’t want my car smelling like weed so I would definitely not smoke in it.” (Urban Woman Non-Consumer 69).*

iii). Peer pressure

Another common factor that was considered by youths when making informed decisions was whether or not their peers would view them less favourably (e.g., being judged as a coward). DUIC was not perceived as dangerous among many youths and refusal to engage in such risky behaviours was believed to hinder one’s social status or alter how one’s character was judged by others.

*I feel like people don’t think much about it, like I would say … if I were to tell my friends that I wasn’t comfortable driving under the influence of cannabis, they would think I was just being, like, soft or something. They don’t think it’s a big deal at all in my school. (Urban Girl Non-Consumer 25).*

It was also noted by young adults that sometimes there was peer pressure to get in the vehicle as opposed to potentially safer alternatives which also posed risks. As one participant stated:

*It’s so easy just to be pressured to get in the car in the moment, because if not … you’ll have to walk home. And honestly, at this point, I don’t even know if it’s safer to walk home in the community. (Rural Woman Consumer 62).*

#### Theme 2: knowledge and awareness

Much of the discussion was premised on an overall lack of awareness of how cannabis impacted driving ability. Specifically, the conversation often centred around: i) the evidence for cannabis and driving, and ii) cannabis versus alcohol.

i). Evidence for cannabis and driving.

Many expressed a lack of empirical evidence to support public health statements that cannabis impairment is similar to that of other elicit substances. As noted by one young adult, *“I guess they take the no news as good news, there are no signs out saying that it’s really bad” (Rural Woman Consumer 81)*.

Participants noted that there was lack of discussion about cannabis-impairment, with minimal reporting of incidences where the accident was contributed to cannabis-impairment. This was said to have led to the idea that if it is not advertised as much as alcohol than it must not be as concerning.

*we have organizations like MADD that show that the really graphic emotional videos of drunk driving, we don’t really have that for cannabis. So, the government [is] … just trying to piggyback off the drunk driving campaign saying the same thing about cannabis. But the information isn’t really out there, at least as graphic and in a way that you remember it as some of the drunk driving campaigns are. (Rural Woman Consumer 36).*

ii). Cannabis versus alcohol

Some suggested that the mixed messaging received in differentiating the overall safety of cannabis compared to alcohol had led people to believe DUIC was safer than after consuming alcohol. As discussed during a youth FG:

*whatever we learn about smoking weed in school, they always say that smoking is safer than drinking alcohol, which is true to an extent. You can get sick from alcohol a lot easier than you can from smoking. You know, there’s a lot more health things that you can get from smoking, from drinking alcohol compared to smoking weed. So, I think that’s the reason why some people have perceived that driving under the influence of cannabis is not bad because we’ve always been taught that, you know, it’s not as bad as drinking alcohol. (Urban Girl Consumer 99).*

#### Theme 3: perception of risk

Perception of risk was a dominant theme throughout the sessions and highlighted misunderstandings regarding in the impacts of cannabis on driving. Key sub-themes that emerged included i) perceived driving ability, and ii) cannabis tolerance.

i). Perceived driving ability

Some individuals who had experienced DUIC themselves or with peers reported enhanced driving abilities following use of cannabis. As one youth noted: “*I know people who say that they think that they’re better drivers when they’re high.” (Urban Girl Consumer 99)* It was also discussed by other participants that the consumption of cannabis prior to driving increased their attentional capacities and had greater awareness of their surroundings.

*I think [people] think that weed or marijuana slows them down. So, they’re going to be more careful where alcohol kind of makes you more impulsive and not as careful. I think that might be the perception that some people have and why they make the conscious decision to drive under the influence of weed. (Rural Woman Consumer 62).*

The frequency of consumption was also believed to be a factor worthy of consideration. Youth and young adults shared that if they or their peers were to DUIC, they felt more comfortable when the individual was a frequent consumer. This was primarily based on the perception that frequent consumers may exhibit increased self-awareness of their driving ability as compared to a non-regular consumer.

*I think the people that are smoking and driving are people that have been smoking for a while and can know they’re high. Like, you kind of know how to handle it … I think that someone’s first or second time smoking and they don’t really know … and then for them to go and sit behind the wheel and like drive, I think they’d kind of be more on edge than someone you know who kind of smokes every day and […*] *that they will have enough consciousness to be able to stay safe on the road. (Rural Girl Consumer 22).*

ii). Cannabis tolerance

The concept of tolerance to cannabis was discussed by some. One person indicated that they were concerned about more risky driving behaviours after cannabis legalization due to the lack of tolerance and awareness of those newer cannabis consumers. There were, however, mixed perceptions from participants regarding their personal trust in driving with some who is an experienced cannabis consumer. One youth spoke stated *I definitely don’t think it’s the same level of danger. I trust people like men who have cars and drive high if they’ve been smoking weed for a while, if they have a lot of experience with it.” (Urban Girl Consumer 76).* Others expressed concern that people justify DUIC because they’re experienced consumers and have built up tolerance.

*I hear a lot of people justify it being like, well, I smoked every day since I was like 10 or 12, so it’s I’m used to it. I drive better. Maybe it’s like a tolerance thing. People used to justify it... I don’t think you actually can build a tolerance that much to something that it’s safe to do still. But that’s what I hear a lot of people sort of backing up the reason for it. (Urban Woman Consumer 37).*

#### Theme 4: mode of transportation

Decisions regarding the choice to DUIC was impacted by modes of transportation. Participants differentiated between i) on-road vs. off-road vehicles and ii) access to transportation.

i). On-road vs. Off-road vehicles

Cannabis-impaired driving was noted to be more common among drivers operating off-road over on-road vehicles. This was discussed in relation to all-terrain vehicles (ATV), boats, and snowmobiles. As one youth shared:

*He actually got ran over on his bike by someone on an ATV who was high. And they had their head down and they completely ran over him and ran him into a ditch because they were high, and they didn’t notice. So, I feel like people driving high on ATVs and dirt bikes,* etc.*, is something I’ve definitely noticed more than people driving behind the wheel of a car. (Urban Girl Consumer 99).*

During a young adult FG, they discussed how driving off-road vehicles offers greater convenience and the belief that less risk is posed to others when DUIC off the road:

*if they’re camping, they could take it over to someone else’s campsite or like that or like to a cabin … I think that more people would not hesitate and then they also might think, well, there’s no one else like on the road or on the trail. So, like, I’m not going to hurt anybody else, it’ll just be me, but you could run into someone else, and you could hurt yourself very easily. (Urban Woman Non-Consumer 69).*

The higher frequency of DUIC while driving an off-road vehicle was premised around even less frequent traffic stops, as shared in conversation with a young adult *“To speak to rural NL, I know that not everywhere has those new testing things, the police don’t have them in smaller settings … because word has gotten out they don’t have equipment here so it’s just a non-issue here” (Rural Non-Binary Young Adult Consumer 77).* This was echoed by another participant who added *“We have a single cop that comes down from [community] only once every week or two and does a run up and back so no one’s ever being caught” (Rural Girl Consumer 22).*

DUIC was also noted as an easier way for non-licensed individuals to reach their destinations after consuming recreational substances. As another young adult noted: *“I remember when I was younger, none of my friends had their license so a couple of guys would take bikes to a party to consume alcohol and cannabis and ride home on their bikes”* (Rural Woman Consumer 62).

Despite the seeming normalization of DUIC, thinking about the potential risks or taking them into consideration was far less common when driving an off-road vehicle than on-road vehicle.

*When alcohol is all we really consumed regularly … skidooing and drinking was very, very common. Now that weed is so much more accessible, it seems like it’s pretty much exactly the same. And if you’re out to a cabin party or something like that and snowmobiling or you’re on an ATV and it’s not even a big deal, it doesn’t seem like [people] even think twice about getting behind the wheel. (Rural Woman Consumer 36).*

ii). Access to transportation

Participants also spoke to difficulties faced when not of legal age to drive, as one youth mentioned: “*Sometimes it might be your only option, if your parents smoke and you need them to bring you somewhere.” (Rural Girl Non-Consumer 19)* Young adults also mentioned that driving after consumption was more convenient as opposed to arranging alternative modes of transportation. As one youth noted: *“There’s people out there that probably conceal it because they don’t want to disturb anyone or like my parents will get mad if I call them or, you know, they don’t want to pay for a cab.” (Urban Boy Non-Consumer 10).*

Another concept that was raised by several people, was the prevailing thought that that driving shorter distances was less risky than longer distances. This was particularly noted by those living in rural communities,

*I grew up in [a rural community] and just the fact that there’s this notion that everything’s so close together. So, I mean, if you’re at a party and someone’s drinking, they’re like, oh, I’ll bring you home. It’s only going to be like two minutes. People, I think, would get in the vehicle and just take that risk. And that’s why there’s been so many incidents of people dying from people under the influence in the small town because people just don’t really see it as they kind of see like everything’s close together. (Rural Woman Consumer 62).*

#### Theme 5: detection

Participants repeatedly referred to a lack of detection for DUIC. This was in reference to detection from both peers and general public as well as law enforcement professionals.

Detection of a peer’s level of cannabis impairment was deemed challenging when compared to alcohol. It was expressed by many that they felt well prepared to recognize signs of alcohol impairment (e.g., smell and behavioural signs), but they lacked insight on how to determine if someone is impaired by cannabis unless the driver disclosed it. As one participant noted, “*I know a lot of guys that I’ve actually driven with when I couldn’t even tell that they were high until like we were like halfway to the stop and then they like mentioned that.” (Urban Girl Non-Consumer 25)* Other youth spoke about the ability for drivers to pretend not be high, which can make it difficult to detect. *It’s like a mental thing where you can kind of snap out of it and pretend to be not high and fool people around you. (Urban Girl Consumer 24).*

Some participants also discussed how cannabis was challenging for the general public to detect drivers who were consuming cannabis. As was discussed by youth in one FG, smoking a joint can look like a cigarette or a vape pen when stopped at a traffic light.

Participants suggested that it was easy to hide impairment from the authorities, especially when people consume products like edibles, that have no distinct smell. As one youth stated: “*… you can smell alcohol on somebody’s breath, but if you eat an edible, you’re not going to smell it. So that’s a problem that they have with it and that’s when they have to use those [breathalyzers]. (Rural Transgender Boy 64).*

There was concern that cannabis was not detected as often as alcohol. *We see routine breathalyzers every now and then. This happened to me a couple of months back, but we don’t see routine swabs for marijuana. It’s a cheek swab … And like I’ve never heard of someone in this area getting ticketed for driving under the influence of marijuana. (Rural Woman Consumer 36).*

The limited presence of police officers in rural areas was also highlighted as a potential contributor to more frequent DUIC. However, participants noted that when the police are present, they’ll stop vehicles and attempt to check for impairment.

### Mapping of road safety themes on the multi-level and systems model of driving behaviour

The primary findings from the identified road safety themes and sub-themes were subsequently mapped onto the multi-level and systems model of driving behaviour using the models classification into antecedents, choice points, and consequences associated with DUIC [32]. Mapping of our primary perception findings onto the multi-level and systems model of driving behaviour supported further sub-classification across the multi-level systems. Exploration of the antecedent perceptions supported further categorization into individual, passenger, and vehicle-related characteristics. Perceptions related to choice points could be further distinguished based on their relation to public policy, enforcement of laws, and opportunities for education. Lastly, perceived consequences could be subdivided into DUIC-related driving behaviours and potential DUIC outcomes (see Table 2).

**Table 2:** Mapping of road safety themes and sub-themes onto the dimensions of the multi-level and systems model of driving behaviour (Adapted from Juarez et al., 2006 [32])

Multi-Level & Systems Model of Driving Behaviour [32]		DUIC and Road Safety Findings from Current Study
**Antecedents**	Individual Characteristics	• Those without a driver’s license were more likely to DUIC using off-road vehicles. • Boys and men were identified to be take on more risky behaviours with cannabis • Perception that frequent cannabis consumers are more tolerant to impairment with greater awareness to compensate for deficits in driving ability.
	Passengers	• Detection by peers is difficult without explicit observation of prior consumption or disclosure by driver • Passengers consume on their own or with the driver • Driver can be exposed to second-hand smoke from passengers • There is peer pressure to accept a ride with a cannabis-impaired driver • Element of trust in their peers or designated drivers.
	Vehicle Characteristics	• Permission to consume cannabis in vehicles is dependent on the rules of the drivers • DUIC was reported as more common with off-road vehicles than on-road vehicles (e.g., ATVs, boats, snowmobiles) • Off-road vehicles are convenient to drive in remote area with lower age requirements for operation • Perception that off-road vehicle pose less risk for harm than on-road vehicles (e.g., less travelled trails or sole operation of a vehicle without passengers).
**Choice Points**	Public Policy	• Lack of evidence for harms associated with cannabis and driving • Lived experience contradicts claims of danger
	Enforcement of Federal & Provincial Laws	• Detection is challenging as cannabis products resemble tobacco products or do not always produce a detectable odour (e.g. edibles). • Detection by law enforcement professionals as impairment expressed as easier to hide with non-odour edibles or present themselves in an incongruent manner for DUIC. • Minimal report of accidents related to cannabis-impairment. • Frequency of police patrolling varies by geographic location (e.g., weekly patrols or less frequent enforcement in rural settings) • Less common experience of impaired driving checks for off-road vehicles than on-road vehicles.
	Educational Community Intervention	• Knowledge and awareness of risks for cannabis versus alcohol • Lack of discussion about cannabis-impairment • DUIC is perceived as safer than driving after alcohol consumption • Perception that cannabis consumption leads to enhanced driving performance.
**Consequences**	Driving Behaviour	• Normalization of DUIC • Social acceptability to DUIC • Peer pressure to drive after consuming cannabis or drive with an impaired driver • Driving and cannabis consumption is a social activity • Passenger with a cannabis-impaired driver • Compensatory driving behaviours (e.g., driving shorter versus longer distances)
	Outcomes (Injury, Death, Costs)	• Harm to oneself with sole operation of a motorized vehicle • Harm to others as passengers in the vehicle, other drivers on the road, or community members sharing the road (e.g., pedestrians) • Negative impact on caregiver relationship (e.g., upsetting a caregiver when requesting late night support) • Threat to social status among peers with refusal to DUIC or accept a ride from a peer who has previously consumed. • Monetary costs incurred to receive transportation from a taxi.

## Discussion

Our study provided insight into the perceptions of youth and young adults about the impact of cannabis consumption on road safety. The facilitated discussions uncovered the perception that DUIC had become a normalized and socially acceptable practice with the potential for further perpetuation in the face of peer pressure. Regardless of age, there appeared to be a lack of knowledge around cannabis and driving risk, with some misconceptions regarding perception of risk. It was commonly shared that cannabis was less dangerous than alcohol and there was a gap in education on the impact of cannabis impairment on driving ability. A number of prominent individual and contextual factors emerged for increased youth in DUIC related behaviours, including a) self-identification as a boy or man, b) the operation of off-road vehicles, c) living in rural areas, and d) limitations with detection of cannabis impairment.

Youth and young adults believed that there is an array of factors that comes into play when emerging adolescents decide to drive when impaired by cannabis. As Juarez et al. (2006) [32] have modelled (see Fig. 1 of Juarez et al., 2006 [32]), the factors can come into play at different periods such as ‘antecedents’ that occur before the engaging in impaired driving, choices that present during operation of a motorized vehicle while under the influence of cannabis, and consequences signified by the magnitude of the potential harms posed to oneself or others that can be influenced by the road conditions (e.g., poor visibility or road conditions) or lack of implementation of safeguards (e.g., frequency of law enforcement in urban versus rural areas). Interventions are important at all levels to prevent or reduce the commonality of DUIC as exemplified by our findings (see Table 2) by tailoring awareness campaigns to the antecedents, equipping youth with knowledge to inform decision making when reaching various ‘choice points’, and minimizing the size of the consequences (i.e., injury or fatalities).

Extending upon this notion, another concerning finding from our study was that DUIC or passenger consumption had become a normative practice in society. Recent data from the *National Cannabis Survey (NCS)* noted 30% of youth (15 to 24) reported DUIC compared to only 16% of adults over 25 [33]. Moreover, past findings from an international survey of youth between the age of 16 to 19 revealed DUIC occurred more frequently among adolescents in Canada than those residing in England [28]. In our study, there was the perception that young males engage in DUIC more than females, which aligns with the NCS with males reporting DUIC twice as often than females [33, 34]. The NCS [33] also identified a small gender difference in risk perception with females found to be more likely to associate DUIC within an hour of consumption to be of graver danger than males perceiving no potential for increased harm [35]. Males were also more likely to vape or consume edibles when driving under the influence of cannabis, leading to later impulsive or erratic driving behaviour on the road or increased risk of accidents [36]. Recent findings by Earle et al. (2019) [37] found that young males tend to report the perspective that DUIC will lead to minimal harm, low levels of impairment, and are more accepting of DUIC than young females. As a result, the lack of perceived harm, consequence, or change to driving ability contributes to the prevalence of youths’ engagement in DUIC. In contrast to past studies, Carpino et al. (2020) [38] did not report any differences in perceptions between genders or whether youth resided in urban versus rural locations.

As a result, it is important that public health campaigns and youth education take into account the gender composition of their target audiences to improve the applicability of the content created for youth. Numerous studies have demonstrated that lower perception of risk is a predictor for DUIC behaviours [24, 29, 39, 40]. Our findings highlight the need for a greater emphasis on educating youth about the dangers posed to themselves and society when choosing to DUIC or ride with a cannabis-impaired driver. Perceptions, especially among youth, are malleable with the potential for efficacious and empirically informed education to promote behaviour change [41]. Furthermore, the development of accessible literature tailored to geographic regions, age groups, and genders brings forth potential to reduce the risk for fatalities or life-changing harm to those at most significant risk, when made applicable or relatable to their everyday lives.

Our study also revealed common misconceptions around perceived risk of the impact of cannabis on driving ability, with many youths and young adults unaware of the impact that cannabis has on one’s driving ability. In some instances, there was the perception that cannabis can enhance driving performance or that experienced consumers had built up a tolerance which mitigated their impairment. Past research has revealed that nearly 75% of Canadians believe cannabis has the potential to harm driving ability [42], however, it has also been noted that cannabis impairment was often perceived as not requiring compensatory behaviour [24]. The *Canadian Cannabis Survey* (CCS) [10] also found complementary results, with 28% of Canadian consumers reporting the effect of cannabis on driving ability was either context-dependent or completely non-existent. Scott et al. (2021) [22, 23] provided further support identifying that beyond context, one of the strongest factors predicting the likelihood to engage in DUIC was the intent to DUIC over the receptibility to drive cannabis-impaired (i.e., planning to DUIC versus being open to the possibility of DUIC).

In our study, DUIC was commonly perceived as less dangerous than alcohol, but recognized that alcohol-impaired driving was never acceptable. These perceptions aligned with Ontario youth, who also reported that DUIC was most likely to occur when cannabis was perceived as low risk, and legalization was supported by society and by younger males [43]. Wickens et al. (2019) [24] also explored perceptions from youth about DUIC and found that denial of risk of harm was common among new drivers, general acceptance among peers, and bias that they were immune to the potential dangers. This is particularly concerning in light of recent findings of a randomized control trial testing youths’ driving performance following consumption of cannabis [44]. They discovered that 100 mg of cannabis consumption by youth did not impact simple driving tasks but led to marked impairment on complex response tasks lasting up to 5 hours. Authors also found that perceived driving ability and safety among youth was significantly lower after cannabis use compared to non-use. This further illustrates the importance of increasing awareness of the impact of cannabis on driving ability, as self-perceived impairment may not be detectable for minor driving performance tasks but can substantially impact response to major operational tasks.

A novel finding from our study was around the reported increased DUIC among drivers of off-road vehicles. The nationwide *Health Behaviour in School-Aged Children survey* conducted pre-legalization (2009-2010) found self-report of DUIC or other substances as the operator or passenger of an on or off-road vehicle to be greatest among youth from rural communities [45]. Although past findings have utilized inclusive definitions of transportation, our study brings forth new support for DUIC as more common with operation of off-road vehicles and the importance of sharing knowledge that can be applied practically by youth to their lived experience (e.g., addressing the perception that driving off-road is a safe alternative to on-road driving). Similar to Pickett et al. (2012) [46], youth in our FGs discussed how off-road vehicles can be driven before they are of legal driving age, while young adults expressed that the off-road vehicle and often the destination are for recreational purposes, tying in the importance of considering intent to DUIC. Hammond and colleagues (2021) [47] also observed similar patterns post-legalization among residents of the Northern territories with significantly higher rates of DUIC than all other provinces in Canada, noting the contribution of the absence of alternative transportation. Youth in our study also spoke to the notion that DUIC was further elevated when residing in rural areas, therefore, extending the impact of rural living on DUIC beyond the Canadian territories.

Youth and young adults in our study also expressed that it was harder to detect cannabis impairment among peers, which could pose safety risk among those who were passengers in vehicles. Many youths felt confident in detecting alcohol but advised that cannabis was easily concealable and regular consumers often did not exhibit signs of impairment. This was also noted in other studies insofar as the absence of efficacious roadside detection methods for cannabis impairment due to the poor reliability of field sobriety [48] and THC blood tests [49, 50]. Instead, Spindle and colleagues (2021) [50] advocated for the employment of a mobile application of executive functioning tasks with field tests for psychomotor and cognitive capability, however, the need for the development of a sensitive cannabis impairment detection method remains ongoing. Improved access to and awareness of cannabis-impairment methods is also important for the protection of public health. As youth and young adults shared in our FGs, they often unknowingly face potential harm when they are unaware of signs of impairment or unable to detect impairment of their designated drivers or others on the road unless it has been explicitly disclosed or prior consumption has been witnessed.

## Limitations & future directions

This qualitative research is not without its limitations. Firstly, many youths (13-18) expressed uncertainty around potential change to road safety post-legalization as they were currently too young to drive or were too young to drive prior to legalization and therefore could not speak to differences. That said, youth were able to speak to the events they have personally witnessed among their social circles or have experienced via direct exposure as a passenger of an individual DUIC. It is also important to consider youth may have spoken in generalized terms or underreported their lived experience to avoid potential involvement with law enforcement should disclose DUIC. Cannabis use, unlicensed operation of a motor vehicle, or DUIC are all illegal activities for their age group. Secondly, DUIC is hard to detect unless explicitly observed on the roadside or can be smelt in the vicinity leading to potential underestimation of how common DUIC is among youth despite frequent DUIC being reported. Facilitation of focus groups with assignment according to past cannabis consumption experience was not feasible as many participants were not of legal age to access cannabis, therefore, it is plausible group differences may exist between consumer and non-consumer youth. Focus groups were instead divided by age into youth (13-18) allowing for comparison of underage youth to of age young adults (19-25), however, it is important to note that the majority of the youth sample (71%) did not have previous cannabis experience. It is plausible that youth in the present study were only able to share their perspective based on their lived experience as a passenger or witness to others who have shared engagement in DUIC. Finally, participants predominantly self-identified as girls or women but reported DUIC happens the most among boys or men. It is plausible that participants’ recollections or perceptions were influenced by their own-gender bias, which may have led to an overestimation of the reported gender difference in DUIC. However, past literature supports DUIC being the greatest among young men in Canada [8].

Future research should aim to integrate the present study’s findings and other perception-based studies to inform the development of cannabis-impaired driving education and awareness content tailored toward youth and young adult drivers as well as passengers of vehicles. Evaluation of education and awareness campaigns should assess the effectiveness in changing behaviour with respect to DUIC and passenger consumption.

## Conclusion

The prominent themes identified in this study highlight concerning behaviours and attitudes among both youth and young adults regarding DUIC. This included the normalization of DUIC, gaps in education, perceptions of risk, modes of transportation, and detection of cannabis. In order to support youths’ decision-making, the development of youth-focused education that will equip youth with decision-making strategies regarding road safety. These findings can be utilized to inform the enhancement of cannabis driving policies to ensure the safety of all citizens.

## Supplementary Information

**Additional file 1.** 

## Data Availability

The datasets generated and/or analysed during the current study are not publicly available due to the sensitive nature of the questions asked, and we are bound by our ethics board to keep data confidential. However, data can be available from the corresponding author on reasonable request following approval from the Interdisciplinary Committee on Ethics in Human Research at Memorial University of Newfoundland.
