# Supplementary material for: Driving under the influence of cannabis: perceptions from Canadian youth
Source: BMC Public Health. 2022 Dec 19;22:2384. doi: 10.1186/s12889-022-14658-9 (PMC9764522; doi:10.1186/s12889-022-14658-9)
Supplement: Supplementary file 1 — Additional file 1. [file 12889_2022_14658_MOESM1_ESM.docx]

Online Supplement 1 for
Driving under the Influence of Cannabis: Perceptions from Canadian Youth (Donnan et al.)

**Semi-Structured Facilitators Discussion Guide for Road Safety**

**Goal:** Explore perceptions about road safety as a result of cannabis use.

**Discussion Questions:**

1. What are your thoughts on people’s understanding of using cannabis in vehicles?
   1. How often are youth driving under the influence of cannabis?
   2. Are passengers in the vehicle using cannabis?
   3. How is using cannabis and alcohol in vehicles different?
2. How has road safety changed since legalization?
3. Have you noticed more tickets or accidents since legalization?
4. How do you feel about road safety? Do you feel more or less safe?
5. What are the differences in how different genders engage with cannabis and driving?
